# April and July Meetings of the Hancock County Medical Association

**Published:** 1860-09

**Authors:** A. Spitler

**Affiliations:** Secretary; Warsaw, Carthage


### APRIL AND JULY MEETINGS OF THE HANCOCK CO. MEDICAL ASSOCIATION.

WARSAW, April 18, 1860.

The Association met pursuant to adjournment. The President being absent the Vice President took the Chair, and called the meeting to order.

Present—Drs. Coolidge, Hofmann, Hall, Hollowbush, Hay and Spitler.

The Association then went into the election of officers by ballot, which resulted as follows:

President—Dr. Charles Hay.

Vice President—Dr. J. K. Boude.

Secretary—Dr. A. Spitler.

Treasurer—Dr. J. W. Hollowbush.

On motion, Drs. Coolidge and Hofmann were appointed delegates to the National Medical Association, which meets in New Haven, in May next, and Drs. Hall and Hollowbush were appointed delegates to the Illinois State Medical Society.

The meeting then adjourned till 1 o'clock, P. M.

AFTERNOON SESSION.

The Association met according to adjournment.

Dr. Hay was then called on, and read a very interesting address on the following subject: "Can the European or Caucasian race arrive at fair physical and intellectual development in hot climates?"

A discussion ensued on the doctrines advanced in the address, which was participated in by Drs. Hollowbush, Hall, Coolidge, and Hay.

Drs. Hofmann, Hollowbush, and Spitler were appointed to prepare and read essays at the next meeting.

The Association, on motion, of Dr. Coolidge, unanimously passed a vote of thanks to Dr. G. W. Hall, for the very faithful and satisfactory manner in which he has discharged the duties of Secretary.

The Association then adjourned to meet in Carthage, the third Wednesday in July next, at 10 o'clock A. M.

A. Spitler, Secretary.

---

Carthage, July 18, 1860.

The Association met according to adjournment.

Present—Drs. Coolidge, Pierson, Hay, Boude, Hall, and Spitler.

An application was made by Dr. D. H. Mason, of Warsaw, to become a member of the Association, which was referred to a committee consisting of Drs. Pierson, Hall, and Spitler, who reported favorably to his reception, which report was adopted.

Cases of interest were then reported by Drs. Pierson, Coolidge, and others.

The Association then adjourned to meet at 1 o'clock P. M.

AFTERNOON SESSION.

The Association met pursuant to adjournment.

On motion, Dr. Coolidge was appointed to deliver an address to the Association at its next meeting.

Drs. Pierson, Hall, Boude, and Spitler, were appointed to read essays at the next meeting of the Association.

Dr. Hay then being called upon, delivered a very interesting address on "The best mode of improving the physical and intellectual condition of man," which was requested of him for publication.

Drs. Hall and Spitler were appointed a committee to have the address published.

On motion of Dr. Spitler, a resolution was passed that this Association memorialize our State Legislature, at its next session, to pass a law requiring the registration of marriages, births, and deaths, in compliance with the late action of the State Medical Association.

On motion of Dr. Coolidge, Drs. Hay and Spitler were appointed a committee to prepare a suitable memorial and present it at our next meeting for action.

On motion, the Association then adjourned, to meet in Carthage, on the third Wednesday in October next.

A. Spitler, Secretary.

